# STAT6-mediated BCL6 repression in primary mediastinal B-cell lymphoma (PMBL)

**DOI:** 10.18632/oncotarget.1149

**Published:** 2013-07-13

**Authors:** Olga Ritz, Karolin Rommel, Karola Dorsch, Elena Kelsch, Julia Melzner, Michaela Buck, Karen Leroy, Vasiliki Papadopoulou, Simon Wagner, Ralf Marienfeld, Silke Brüderlein, Jochen K. Lennerz, Peter Möller

**Affiliations:** ^1^ Institute of Pathology, University Ulm, Ulm, Germany; ^2^ Institute for Research in Biomedicine, Bellinzona, Switzerland; ^3^ INSERM, U955, and Université Paris Est Créteil, Hôpital Henri Mondor, AP-HP, Département de Pathologie, Créteil, France; ^4^ Department of Cancer Studies and Molecular Medicine and MRC Toxicology Unit, University of Leicester, Leicester, UK

**Keywords:** PMBL, intratumoral heterogeneity, BCL6, pSTAT6

## Abstract

Primary mediastinal B-cell lymphoma (PMBL) is characterized by aberrant activation of JAK/STAT-signaling resulting in constitutive presence of phosphorylated STAT6 (pSTAT6). In primary PMBL samples pSTAT6 is only expressed in a sub-population of lymphoma cells in a pattern that is reminiscent of that of the BCL6 oncogene. Double-fluorescence staining was carried out to determine the association between these two proteins in ten primary PMBL cases and three available PMBL cell line models. Surprisingly, only a minute fraction of double-positive nuclei was observed, while each sample contained considerable fractions of single-positive pSTAT6 and BCL6 nuclei. The intratumoral coexistence of BCL6+/pSTAT6− and BCL6−/pSTAT6+ subpopulations suggests a negative interaction between these factors. *In silico* screening of the *STAT6* /*BCL6* promoters for DNA consensus binding sites identified five STAT-binding-sites in the *BCL6* promoter. We confirmed STAT6 binding to the *BCL6* promoter *in vitro* and *in vivo* by band shift / super shift assays and chromatin immunoprecipitations*.* Using *BCL6* luciferase reporter assays, depletion of STAT6 by siRNA, and ectopic overexpression of a constitutive active STAT6 mutant, we proved that pSTAT6 is sufficient to transcriptionally repress BCL6. Recently developed small molecule inhibitors 79-6 and TG101348 that increases BCL6 target gene expression and decreases pSTAT6 levels, respectively, demonstrate that a combined targeting results in additive efficacy regarding their negative effect on cell viability.

The delineated pSTAT6-mediated molecular repression mechanism links JAK/STAT to BCL6-signaling in PMBL and may carry therapeutic potential.

## INTRODUCTION

Primary mediastinal B-cell lymphoma (PMBL) is a distinct subtype of diffuse, large B-cell lymphoma (DLBCL) that arises in the thymus [reviewed in 1]. While the gene expression signature of PMBL is distinct and differs from other DLBCL subtypes [2;3], morphology and immunophenotype, however, are less characteristic. Originally PMBL was recognized in the 1980s [4;5]; however, it was the recurrent chromosome 9p gains and *JAK2* amplifications that established PMBL as a genetically defined lymphoma entity [1].

**Figure 1:**
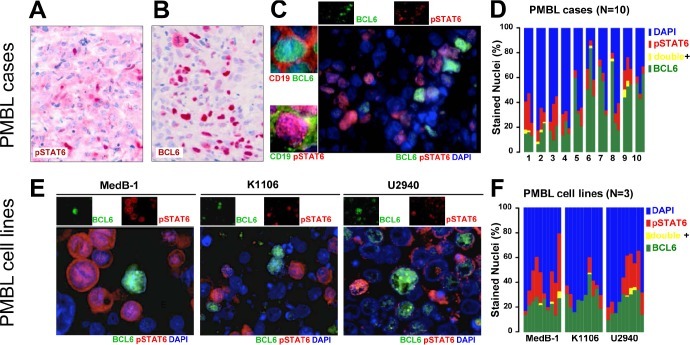
Intratumoral heterogeity of BCL6 and pSTAT6 in PMBL IHC staining for pSTAT6 (A) and BCL6 (B) in ten PMBL tumor samples (representative cases) analyzed on FFPE sections using alkaline phosphatise/RED system. Nuclear distribution of BCL6 and pSTAT6 in PMBL tumors analyzed on snap frozen tumor samples (N=10, representative case is shown) (C) or on FFPE sections of cell pellets of PMBL cell lines, (E) using double IF. Individual channels (upper insets) and co-labeling with the B-cell marker CD19 are provided (left insets). Cytofluorogram shows percent of BCL6+/pSTAT6- nuclei (green), BCL6−/pSTAT6+ nuclei (red), BCL6+/pSTAT6+ nuclei (yellow) or BCL6−/pSTAT6- (blue) nuclei in PMBL cases (D) or PMBL cell lines (F). Bar diagram (D) shows 10 PMBL cases evaluation of 3 optical field each, bar diagram (F) shows 3 PMBL cell lines with evaluation of 10 optical fields each.

PMBL does not harbor JAK2 activating mutations [6]. Therefore, JAK2 signalling may be activated either due to gene-dosage effect of *JAK2* [7] or other molecular aberrations which take place in PMBL. We have shown that frequent occurrence of silencer of cytokine signaling 1 (*SOCS1*) mutations impair proteasomal degradation of phosphorylated JAK2, which prolongs its action and contributes to constitutively activated JAK/STAT signaling in PMBL [8;9]. According to Guiter [10], nuclear pSTAT6 is a characteristic finding in PMBL. However, the extent of nuclear staining is heterogeneous within each PMBL case, and the number of positively labeling neoplastic cells ranges from 10-50% [10]. While the specific functions of pSTAT6 in PMBL are currently unknown [10], we found the heterogeneous nuclear labeling pattern particularly intriguing (Figure 1A) because it reminded us of BCL6 labeling pattern, which, in PMBL, is also micro-heterogenous (Figure 1B) [11-13]. To note, BCL6 is constitutively expressed in ~40% of DLBCL and its overexpression in murine B-cells is sufficient for the development of a disease similar to human DLBCL [14-16]. Although prognostic differences associated with BCL6 content have been reported [17], the relevance of this intratumoral heterogeneity in PMBL has not yet been addressed.

Here we used co-staining of BCL6 and pSTAT6 as a starting point for a combined immunohistochemical, cell-biological and small-molecule inhibitor study. We describe that phenotypically distinct subpopulations coexist in PMBL. We delineated a novel molecular repression mechanism that links activated JAK/STAT signaling to BCL6 expression. Thus, in conjunction with the additive efficacy of small molecule inhibitors on cell viability our findings may carry therapeutic potential.

## RESULTS

### Presence of pSTAT6 and BCL6 is almost perfectly mutually exclusive in PMBL

We quantitatively examined ten primary PMBL tumor samples by double IF for pSTAT6 and BCL6 and verified staining in tumor cells using the B-cell surface marker CD19 (Figure 1C, left cutouts). When compared by fields within and between cases a certain degree of heterogeneity was apparent; however, the fraction of BCL6+/pSTAT6+ nuclei was always minute(0.8±0.2%) and BCL6+/pSTAT6− and BCL6−/pSTAT6+ fractions appeared as distinct subpopulations. Despite a marked case to case variability the average BCL6+/pSTAT6− and BCL6−/pSTAT6+ fractions accounted to 35±3.9% and12±1.5%, respectively (Figure 1D). Of note, averages of BCL6+/pSTAT6− and BCL6−/pSTAT6+ fractions surmount to ~46% of the tumor cells in primary PMBL cases leaving ~54% CD19+ BCL6−/pSTAT6− cells. (Figure 1D). Next, we asked whether this pattern is preserved in currently available cellular models of PMBL and, therefore, examined MedB1[18], K1106[19], and U-2940[20] cell lines using methods described above (Figure 1E). Despite marked field-to-field heterogeneity (Figure 1F), the fraction of BCL6+/pSTAT6+ nuclei (0.61 +/− 0.26%) was also minute in PMBL cell lines. The BCL6+/pSTAT6− fraction (23.9+/−1.5%) and BCL6−/pSTAT6+ fraction (13.9+/−2.3%) surmounted to ~38%.

These data have two implications: first, the very closed to mutual exclusive pattern of BCL6 and pSTAT6 in PMBL suggest their negative interaction; second, PMBL cell lines, showing similar to PMBL tumors pattern of pSTAT6 and BCL6, are suitable cellular models for molecular studies on the negative interplay between BCL6 and pSTAT6.

### pSTAT6 transcriptionally represses BCL6

**Figure 2:**
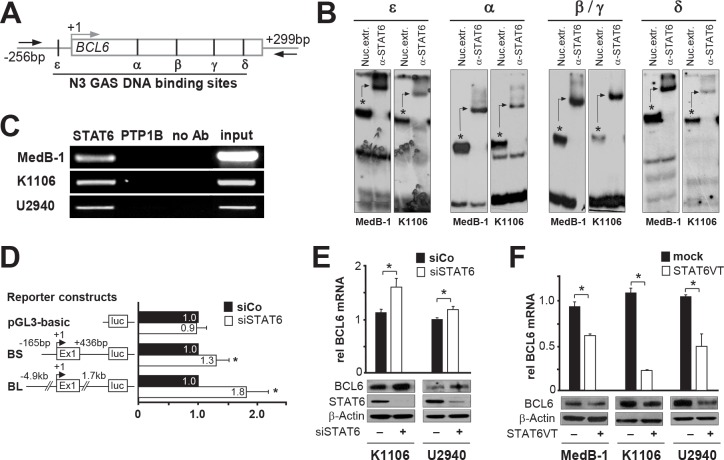
STAT6 represses BCL6 in PMBL (A) BCL6 proximal promoter region, containing untranslated exon 1 (shown as a bar) with five GAS DNA-binding sites (alpha, beta/gamma, delta, epsilon); arrows indicate primers used for ChIP. (B) *In vitro* binding of STAT6 to GAS sites within the BCL6 promoter region in MedB-1 and K1106 was analyzed using band shift- and super shift assays. Asterisks indicate protein/DNA-complex (band shift), which is shifted (arrows) when pre-incubated with anti-STAT6 antibody (super shift). (C) *In vivo* binding of STAT6 to BCL6 promoter region in MedB-1, K1106, and U-2940 was analyzed by ChIP using primers shown in (A). Samples incubated either with anti-STAT6 antibody (STAT6), without antibody (no Ab) or with a control unrelated antibody (PTP1B). Total chromatin input was used as a positive control in PCR (input). (D) Luciferase reporter assays were done in K1106 cells co-transfected with control siRNA (siCo), siRNA targeted STAT6 (siST6) and pGL3 basic vector, BCL6 short (BS) and long (BL) reporter constructs. Results are expressed as relative fold-change of luciferase activity in siST6 relative to siCo. (E) Relative BCL6 mRNA - and protein expression levels in K1106 and U2940 cell lines after treatment with either siRNA specific for STAT6 (siSTAT6) or control siRNA (siCo). The efficiency of STAT6 depletion by siRNA was checked by western blot using STAT6 antibody (middle panel). Beta (β)-actin was used as a loading control for all western blot experiments. (F) Relative BCL6 mRNA - and protein expression levels in all PMBL cell lines after ectopic expression of constitutive active mutant of STAT6 (STAT6VT) or empty vector (mock). All data displayed as bar-graphs provide as averages ± standard error of the mean (SEM) from three independent experiments and p-value indicators * are provided when *p*<0.05.

Based on the staining pattern we considered two possible negative regulatory mechanisms: pSTAT6 represses BCL6 or vice versa. Therefore, we first bioinformatically screened the promoter region of *BCL6* and *STAT6* with regards to specific DNA binding sites for STAT6 [reviewed in 21] and BCL6 [22], respectively. The *STAT6* promoter, including 2kb upstream sequences, revealed no BCL6 consensus DNA binding motives. In contrast, the regulatory region of *BCL6*, including the promoter and the first untranslated exon, contained four known STAT DNA binding sites: alpha, beta, gamma, delta [23;24]. In addition, we identified one previously unrecognized Interferon-gamma activation (GAS)-site upstream of the first exon of *BCL6* and called it epsilon (from -128 to -120bp; Figure 2A). Although a direct link between pSTAT6 and BCL6 has not been reported, based on our promoter sequence analysis we focused our experiments on a possible repression of *BCL6* by pSTAT6.

The *in vitro* binding of STAT6 using electrophoretic mobility shift assay was shown for all five GAS sites, including the newly detected one (EMSA; Figure 2B). DNA-protein-complex (Figure 2B, asterisk) was completely shifted by incubation with anti-STAT6 antibodies (Figure 2B, arrows), indicating the presence of STAT6 in this DNA-protein-complex. The *in vivo* binding of STAT6 to the *BCL6* proximal promoter was assessed by ChIP using a STAT6 antibody. Subsequent PCR amplification indicated specific binding of STAT6 to the regulatory *BCL6* element whereas control samples (precipitated with antibodies against an unrelated cytoplasmic protein PTP1B or without antibodies) did not yield amplification products (Figure 2C). Together, these *in vitro* and *in vivo* findings indicate that STAT6 binds to the examined *BCL6* regulatory region in PMBL cells.

To determine whether STAT6 can repress transcription through interaction with the *BCL6* regulatory regions [23-25], we created two *BCL6* reporter constructs. We cloned (1) the *BCL6* promoter region including first untranslated exon (-165bp to +436bp, called BCL6short =BS); or (2) a 6.5kb portion of the *BCL6* locus, from -4.9kb to +2.0kb relative to transcription start site including the first intron, (called BCL6long=BL) upstream of a luciferase reporter gene [26]. We performed a luciferase reporter assay in K1106 cells where STAT6 was depleted by siRNA against STAT6 (siSTAT6) compared to the control sample (transfected with control siRNA=siCo). Depletion of STAT6 in K1106 cells resulted in a slight increase of luciferase report when the BS reporter construct was used (fold change siST6 1.3 vs. siCo 1.0 ±0.2, *p*=0.05) (Figure 2D). Taking into account that several research groups showed the importance of genomic environment in transcriptional regulation of *BCL6* [23-25;27], we used the BCL6 long (BL) reporter construct [26] and observed clear re-activation of transcription from *BCL6* promoter after STAT6 was knocked down (fold change siST6 1.8 vs siCo 1.0 ±0.3, *p*=0.04) (Figure 2D). In sum, the results of these luciferase reporter assays indicated that STAT6 represses *BCL6* in PMBL and that this repression occurs through the proximal promoter region as well as through the first intron.

Next, we examined whether STAT6 knock-down has a positive effect on BCL6 mRNA and protein expression in K1106 and U-2940 cells. We excluded MedB-1 cells since we previously showed that this cell line is STAT6 dependent [28] and, therefore, inapplicable for this type of experiment. Efficient reduction of STAT6 protein was observed in K1106 and U-2940 after cells were treated with siSTAT6 compared to sample treated with siCo (Figure 2E, middle panel). BCL6 mRNA (Figure 2E, bar graph) and protein (Figure 2E, upper panel) levels were moderately but significantly increased in K1106 and U-2940 samples which were transfected with siSTAT6.

In order to check whether pSTAT6 mediated repression mechanism is operative also in MedB-1, we ectopically expressed constitutive active STAT6 mutant[29], called STAT6VT, in all three PMBL cell lines and analyzed levels of BCL6 mRNA (Figure 2F, bar graph) and of BCL6 protein (Figure 2F, upper panel). In all three PMBL cell lines we observed a significant decrease in BCL6 mRNA and protein amounts in samples where STAT6VT was overexpressed. Our results indicate that pSTAT6 is sufficient to repress *BCL6* in PMBL and that this repression mechanism works in all cellular models of PMBL.

### Small molecule inhibitors against JAK2 (TG101348) and BCL6 (79-6) leads to additive growth inhibition in vitro

**Figure 3:**
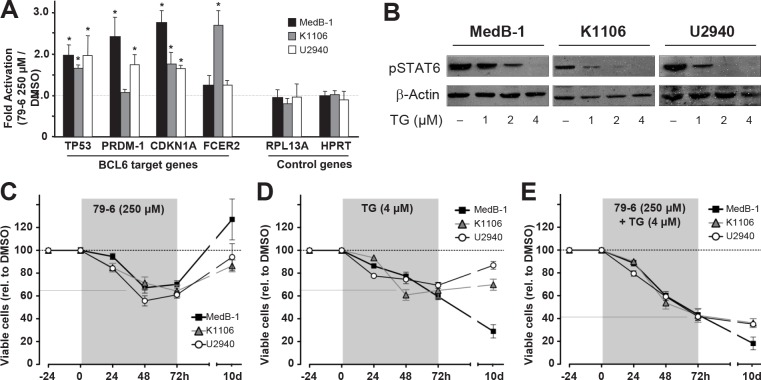
Combined treatment of PMBL cell lines with inhibitors against JAK2 (TG101348) and BCL6 (79-6) results in additive efficacy in cell viability assays (A) Relative mRNA abundance of the BCL6 target genes, TP53, PRDM-1, CDKN1A, and FCER2, and not BCL6 targets, RPL13A and HPRT, measured in MedB-1, K1106, and U2940 cells which were exposed to vehicle (DMSO) or 250μM of 79-6 for 8h. The Y-axis shows 79-6-mediated fold change of each gene expression normalized to HPRT/β-actin and relative to vehicle (DMSO) indicated by a dotted line. (B) Western blot shows dose-depended reduction of STAT6 phosphorylation (pSTAT6) in PMBL cell lines after treatment with the JAK2 kinase inhibition TG101348 for 24h. (C-E) Cell viability assay analyzed by tryphan blue exclusion method after treatment of PMBL cell lines with BCL6 inhibitor 79-6 (250µM) (C), JAK2 inhibitor TG101348 (1µM) (D) or both inhibitors (E) for indicated time (X-axis). Inhibitors were added once (time point 0) and cell viability analysis was performed at time points 24h, 48h, and 72h (highlighted in grey). Then the medium containing inhibitors was replaced with standard medium, cells were incubated for additional 7 days (non-highlighted area) and last cell viability analysis was performed at time point 10 days (10d). The amount of viable cells was calculated relative to corresponding sample treated with vehicle (DMSO), set to 100%, and plotted as dotted line. The solid line shows the average viability decrease of all three PMBL cell lines.

The delineated repression mechanism is associated with the observed mosaic pattern of PMBL and, thus, existence of at least four phenotypically different cellular subtypes of PMBL: BCL6−/pSTAT6+, BCL6+/pSTAT6−, BCL6+/pSTAT6+, and BCL6−/pSTAT6− (Figure 1C-F). BCL6 and pSTAT6 are oncogenic factors, and both have been shown to contribute to tumor cell viability [21;30;31]. To test whether viability of entire tumor cell population is dependent on BCL6 and /or pSTAT6 functions within a subset of cells [32], we applied a cellular assay using recently developed specific inhibitors. First, we adapted BCL6 specific inhibitor (compound 79-6) conditions to PMBL cellular model and established IC-50 value (250µM) (data not shown), which was the same as used for treatment of primary DLBCL tumors [15]. Since the 79-6 compound does not affect BCL6 protein levels but inhibits its repressor properties [15], we controlled the function of 79-6 in PMBL cell lines by analyzing mRNA expression of BCL6 target genes (Figure 3B). Generally, we detected de-repression of BCL6 targets gene (Figure 3B, TP53, PRDM-1, CDKN1A, and FCER2) although slight differences between cell lines were observed. While in MedB-1 and U-2940 cells all analyzed BCL6 target genes were upregulated after treatment with 79-6 (fold activation in MedB-1 cells ranged from1.3 to 2.7; in U-2940 from1.3 to 2.1), we observed no changes in PRDM-1 mRNA expression in K1106. Nevertheless, four other BCL6 target genes were clearly upregulated also in K1106 (fold activation ranged from1.8 to 2.6). In contrast, genes that were not BCL6 targets (Figure 3B, RPL13A, HPRT) were not affected by BCL6 inhibitor and their levels did not change in samples treated with 79-6 peptide compared to untreated cells.

Constitutive STAT6 activation in PMBL was at least partially linked to increased activity of JAK2 [10] due to its genomic amplification characteristic for PMBL [33]. We tested the specific JAK2 inhibitor TG101348 [7] function by analysis of the pSTAT6 protein amount in PMBL cell lines (Figure 3A). We observed a dose dependent decrease of pSTAT6 amount in all PMBL cell lines, and a concentration of 1µM was chosen according to Figure 3A and previously published data [7].

Next, we treated all PMBL cell lines either with BCL6 inhibitor 79-6, which was likely to target BCL6+/pSTAT6- cells (Figure 3C), or with JAK2 inhibitor TG101348, which targets BCL6−/pSTAT6+ cells (Figure 3D), or with both inhibitors, to target both cell populations (Figure 3E). Inhibitors were added once and cell viability was analyzed by trypan blue exclusion at time points 24h, 48h, 72h (Figure 3C-E, highlighted in gray). After 72h incubation with inhibitors cell culture medium was changed and cells were incubated for additional week in medium without inhibitors. Last analysis of cell viability was done at day 10 after adding inhibitors (Figure 3C-E, not highlighted).

The BCL6 inhibitor 79-6 at time point 72h reduced cell viability in all three PMBL cell line by about 30% (taken the mean between three cell lines) (Figure 3C). Of note, one week after removal of 79-6 inhibitor, cell viability in all PMBL cell lines was restored, supporting the reversibility of 79-6 effects as previously reported [15]. Inhibition of JAK2 and subsequent reduction of pSTAT6 resulted in reduction of cell viability also in all PMBL cell lines by about 35% (taken the mean between three cell lines) (Figure 3D). Interestingly, after TG101348 was removed and cells were cultured for additional week, only K1106 and U-2940, but not MedB-1, were able to regenerate their viability. This observation confirms our previous data that MedB-1 depends on pSTAT6 [28]. The combination of two inhibitors, targeting both populations, BCL6+/pSTAT6- as well as BCL6−/pSTAT6+, lead to an additive effect, and cell viability was reduced by about 65% in PMBL cell lines (taken the mean between three cell lines) (Figure 3E). Culturing of PMBL cell lines for an additional week without inhibitors did not result in the recovering of cell viability, but showed a sustained reduction of it (Figure 4E, not highlighted region). Therefore, our results show that targeting both co-existing populations in PMBL cell lines had an additive and prolonged effect compared to each single inhibitor.

## DISCUSSION

Immunohistochemistry for BCL6 is a part of the routine diagnostic work-up for B cell lymphoma, whereas pSTAT6 was suggested to be a marker for discriminating the PMBL group from other DLBCL subtypes [10]. Heterogeneous expression of both transcription factors in PMBL was previously reported [10-13]. Furthermore, a regulatory link between several STAT family members and BCL6 was already observed in normal B lymphocytes. For instance, Schroder et al. observed decreased BCL6 transcript levels after STAT6 depletion in IL-4 stimulated murine B lymphocytes [34] while STAT5 has been shown to upregulate BCL6 in human CD19 positive B cells [35]. Collectively, these data suggest that STATs are positive regulators of BCL6 expression in B lymphocytes. Surprisingly, we observed an almost perfect mutual exclusive pattern of pSTAT6 and BCL6 in PMBL patient samples and cell lines by analyzing BCL6 and pSTAT6 presence at the same time using double IF. Thus, our observation led us to assume a negative mutual interference of activated pSTAT6 and BCL6.

STAT factors are latent and localized in the cytoplasm until activation of a signalling pathway, which is usually initiated upon binding of cytokines to their receptors, leads to the activation of JAK kinases, finally resulting in the phosphorylation of STAT monomers. Phosphorylated STAT monomers dimerize and translocate to the nucleus where they control the expression of a distinct set of target genes. In contrast to this scenario observed in non-malignant B cells, constitutively activated and nuclear localized STATs were reported in different hematopoietic and nonhematopoietic cancers [36]. Several molecular mechanisms are known to take part in the constitutive activation of the JAK-STAT pathway including the genetic aberrations of negative regulators like *SOCS-1* or the amplification of the *JAK2* gene. Importantly, the signaling leading to a constitutive activation of the STATs might also impinge the function of these transcription factors. For instance, in contrast to its positive role in CD19+ B lymphocytes, STAT5 has been shown to repress BCL6 in B cell lymphoma [23]. In consequence, a functional modification due to a constitutive activation of the JAK-STAT pathway might also affect and modify the STAT6 function in a way that it acts as a repressor in PMBL, whereas in murine B lymphocytes it seems to activate BCL6 in response to IL-4 induction [34].

Several previous studies suggested STAT6 as a transcriptional repressor in human and murine lymphocytes [34;37-39], although in B cells BCL6 was not reported as a target for STAT6 repression yet. Baus et al. and Schroder et al. depleted STAT6 in classical Hodgkin lymphoma cell lines and in IL-4 stimulated murine B lymphocytes, respectively. In both studies, the number of upregulated genes after STAT6 depletion was higher than that of downregulated ones [34;37]. Thus, repressive properties might be a general feature of STAT6. However, the target genes affected by such a STAT6-mediated repression may differ between normal B lymphocytes and B lymphoma cells.

Another important question concerns the molecular mechanisms underlying the repressive effect of STAT6 in lymphoma cells. One of such molecular mechanisms could be the mutation in the DNA-binding domain (DBD) of STAT6 which we recently identified in 36% of PMBL [40]. These mutant STAT6 proteins display an altered DNA binding ability which might explain a functional difference regarding its impact on BCL6 expression [40]. However, among the PMBL cellular models used in this study only MedB-1 cells harbour heterogeneous STAT6 missense mutations [40], whereas in K1106 [40] and in U-2940 (own unpublished data) STAT6 is wild type. Nevertheless, the repressive effect was eminent in all PMBL models suggesting that mutations in the DBD of STAT6 might not be the reason for its repressive effect on BCL6 expression. However, the STAT6 knock down experiments confirmed our previous results that only MedB-1 cells are STAT6 dependent [28] and potential role of STAT6 mutations for this dependency should be addressed in future studies.

We showed that pSTAT6 represses BCL6 expression by binding to several GAS DNA binding sites in the regulatory region of *BCL6* (Figures 2C and 2B). While the existence of these GAS elements in the promoter region of *BCL6* and their occupancy by pSTAT6 is a clear basis for the pSTAT6 mediated *BCL6* repression, the mechanism of pSTAT6 reduction by BCL6 still remains elusive. There are no perfect BCL6 binding sites [22] within a 2kB upstream region of *STAT6*. However, the existence of one GAS element (N4) at the position -192 to -183 relative to *STAT6* transcription start (+1) in conjunction with the ability of BCL6 to bind to GAS elements although with low affinity [22;41] prompted us to test whether BCL6 could attenuate STAT6 expression. However, after depletion of BCL6 by siRNA and analysis of STAT6 mRNA-expression levels, we failed to find evidence in support of such a repression (data not shown). Hence, it remains to be determined what factor(s) orchestrates the absence of pSTAT6 in the BCL6-positive cells. However, an indirect mechanism affecting the JAK2 mediated phosphorylation and/or the PTPN1 mediated dephosphorylation of STAT6 may be at work. Additionally, secondary regulatory factors and miRNA-induced STAT6 degradation could cooperate and maintain absence of STAT6 in a subset of PMBL cells.

Importantly, the double-negative population present in PMBL cases and cell lines appears to be driven by other survival and growth pathways then BCL6 and pSTAT6 signallings. One of major aberrantly activated pathway in PMBL is NF-κB signaling [2;42]. Therefore, it will be interesting to determine whether the NF-κB signalling pathways specifically drive the BCL6−/pSTAT6- population.

Observed intratumoral phenotypical heterogeneity in PMBL and delineated repression mechanisms lead us to present two hypothesises with regards to PMBL tumor pathophysiology: First, an intratumoral “multi-dependency” may exist in PMBL which protects the tumor from addiction to one oncogene [32;43;44] and thus provides a higher flexibility regarding the adaption to tumor environment [45]. This notion is supported by the results of our inhibitor assays: in all PMBL cellular models each single inhibitor affected only a part of cells. Of note, this fraction is always equivalent to the fraction containing the targeted molecule. Moreover, the combination of two inhibitors had no synergistic but only additive effects suggesting that no cell subtype is dominant over the other in determining survival.

Our second hypothesis is based on the cancer stem cell (CSC) theory [46;47], which implies that differentiation of CSC leads to the production of all cell types in the tumor and, therefore, generates intratumoral heterogeneity. Thus, the four phenotypically different cell subtypes observed in PMBL may represent tumor cells in different development states. Arguing along this line, the few BCL6+/pSTAT6+ cells may be those caught at the moment where pSTAT6 starts acting as BCL6 repressor.

Since the implications and clinical importance of intratumoral diversity have been widely accepted, the number of studies assessing intratumoral heterogeneity caused by genetic and non-genetic process in tumors like breast cancer, non-small-lung cancer, cervical cancer and many others [48] increases rapidly. To our knowledge, in the lymphoma field this phenomenon was not addressed so far. The main future challenge for the treatment of PMBL is the identification of patients which will either show a relapse or which are resistant to current therapies. Here, further analysis and characterization of cell subtypes in PMBL may help to improve the prognosis of PMBL patients [42].

In sum, our present work shows a novel function of constitutive activated STAT6 in PMBL and provides insights into pathophysiology of PMBL. We suggest that targeting specific sub-population of lymphoma cells defined by expression of particular transcription factors could be helpful in developing of new clinical approaches to this lymphoma.

## METHODS

Details of the Design and Methods are available as Supplement

### Ethics Statement

Investigation has been conducted in accordance with the ethical standards and according to the Declaration of Helsinki and according to national and international guidelines and has been approved by the author's institutional review board.

### PMBL patient samples and immunostainings

The PMBL cases were identified retrospectively, anonymized, and approval from the local ethics committee was obtained. BCL6 and/or pSTAT6 were stained using immunohistochemistry or immunofluorestence (IF) on formalin-fixed or snap frozen samples (PMBL patients and cell lines) using antibodies against BCL6 (#M7211; 1:20; Dako, Aachen, Germany) or pSTAT6 (Tyr641; #cs-9361 at 1:30; New England Biolabs, Frankfurt am Main, Germany). BCL6 and pSTAT6 markers were evaluated in conjunction with DNA labeling using a quantitative image analysis as previously described [49].

### Cell culture, transfection, and siRNA treatment

Three established human PMBL cell lines MedB-1, K1106, and U-2940 were maintained as described [8]. PMBL cell lines were nucleofected using the manufacturer's dedicated protocol (Amaxa, Cologne, Germany) and followed conditions: for MedB-1- bufferV, program U-01 (vector) R-01 (siRNA); for K1106- buffer T, program O-20 (vector) N-20 (siRNA), for U-2940-buffer T, program X-05 (vector/siRNA). Control siRNA (siCo; #VC300A2; Sigma-Aldrich,Taufkirchen, Germany) or siRNA targeting STAT6 (#STAT6VHS41762, Life Technologies, Darmstadt, Germany) were nucleofected into PMBL cells (0.2nmol per sample). Cells were harvested 72h post transfection for all further experiments. Ectopic expression of constitutively active STAT6 (STAT6VT)[29] was performed using 1µg of pcDNA-control plasmid or pcDNA plasmid containing STAT6 sequence with activated mutations. Cells were harvested 48h post transfection for all further experiments.

### Western blot, band-shift, and super-shift assays

Western blot, band shift-, and super shift assays were performed as described [28;40]. Briefly, we used 10µg total extracts per lane and antibodies against BCL6 (N3 #sc-858); STAT6 (M-200 #sc-1698, Santa Cruz, Heidelberg, Germany); pSTAT6 (Tyr641; #cs-9361 New England Biolabs) for western blot. For band shift- and super shift assays we used 1µg nuclear extract and 1µg anti-STAT6 (#sc-621, S-20X; Santa Cruz) antibody.

### Chromatin immunoprecipitation (ChIP)

The ChIP Assay kit (#17-295, Millipore, Schwalbach, Germany) was used according prior protocols [50]. We used 1µg antibodies against STAT6 (sc#1698, M-200, Santa Cruz) for immunoprecipitation.

### Luciferase reporter assays

Luciferase reporter assays were described previously [28;40]. K1106 cells were transfected with siCo or siSTAT6 and after 48h incubation cells were additionally transfected with reporter constructs: pGL3-basic vector (Promega, Mannheim, Germany), *BCL6* short construct (-165bp to +436bp), and *BCL6* long construct[26].

### QuantitativePCR (qPCR)

2µg of total RNA were reverse transcribed into cDNA using Superscript II kit (Life Technologies) and amplified using the SYBR Green master mix on an iCycler (BioRad). Each PCR reaction was performed in triplicate and average Ct values were calculated using the 2(-∆∆C(t)) method [51]. Gene expression levels are provided relative to the reference genes analyzed by geNorm software (http://medgen.ugent.be/~jvdesomp/genorm/; (last accessioned Nov 2011)[52].

### Drug treatment and cell viability assays

PMBL cells were treated once either with JAK2 inhibitor (TG101348) (1µM) or with BCL6 inhibitor (79-6) (250µM) or with both inhibitors. Cell viability, determinated by Trypan Blue exclusion, was performed in 24h increments. After 72h the medium was replaced by medium without inhibitors and samples were incubated for an additional week (timepoint 10 days).

### Statistics

For statistical comparisons we used t-tests, ANOVA and Fisher's exact test. Statistical significance was defined as *p* <0.05.

## SUPPLEMENTARY METHODS



## References

[1] Swerdlow SH, Campo E, Harris NL, Jaffe ES, Pileri SA, Stein H, Thiele J, Vardiman JW (2008). World Health Organization Classification of Tumours of Hematopoietic and Lymphoid Tisues.

[2] Feuerhake F, Kutok JL, Monti S, Chen W, LaCasce AS, Cattoretti G, Kurtin P, Pinkus GS, de,Leval L, Harris NL, Savage KJ, Neuberg D, Habermann TM, Della-Favera R, Golub TR, Aster JC, Shipp MA (2005). NFkappaB activity, function, and target-gene signatures in primary mediastinal large B-cell lymphoma and diffuse large B-cell lymphoma subtypes. NFkappaB activity, function, and target-gene signatures in primary mediastinal large B-cell lymphoma and diffuse large B-cell lymphoma subtypes.

[3] Rosenwald A, Wright G, Leroy K, Yu X, Gaulard P, Gascoyne RD, Chan WC, Zhao T, Haioun C, Greiner TC, Weisenburger DD, Lynch JC, Vose J, Armitage JO, Smeland EB, Kvaloy S (2003). Molecular diagnosis of primary mediastinal B cell lymphoma identifies a clinically favorable subgroup of diffuse large B cell lymphoma related to Hodgkin lymphoma. Molecular diagnosis of primary mediastinal B cell lymphoma identifies a clinically favorable subgroup of diffuse large B cell lymphoma related to Hodgkin lymphoma.

[4] Moller P, Lammler B, Eberlein-Gonska M, Feichter GE, Hofmann WJ, Schmitteckert H, Otto HF (1986). Primary mediastinal clear cell lymphoma of B-cell type. Primary mediastinal clear cell lymphoma of B-cell type.

[5] Moller P, Moldenhauer G, Momburg F, Lammler B, Eberlein-Gonska M, Kiesel S, Dorken B (1987). Mediastinal lymphoma of clear cell type is a tumor corresponding to terminal steps of B cell differentiation. Mediastinal lymphoma of clear cell type is a tumor corresponding to terminal steps of B cell differentiation.

[6] Melzner I, Weniger MA, Menz CK, Moller P (2006). Absence of the JAK2 V617F activating mutation in classical Hodgkin lymphoma and primary mediastinal B-cell lymphoma. Absence of the JAK2 V617F activating mutation in classical Hodgkin lymphoma and primary mediastinal B-cell lymphoma.

[7] Rui L, Emre NC, Kruhlak MJ, Chung HJ, Steidl C, Slack G, Wright GW, Lenz G, Ngo VN, Shaffer AL, Xu W, Zhao H, Yang Y, Lamy L, Davis RE, Xiao W (2010). Cooperative epigenetic modulation by cancer amplicon genes. Cancer Cell.

[8] Melzner I, Bucur A.J, Bruderlein S, Dorsch K, Hasel C, Barth TF, Leithauser F, Moller P (2005). Biallelic mutation of SOCS-1 impairs JAK2 degradation and sustains phospho-JAK2 action in the MedB-1 mediastinal lymphoma line. Blood.

[9] Melzner I, Weniger MA, Bucur AJ, Bruderlein S, Dorsch K, Hasel C, Leithauser F, Ritz O, Dyer MJ, Barth T F, Moller P (2006). Biallelic deletion within 16p13.13 including SOCS-1 in Karpas1106P mediastinal B-cell lymphoma line is associated with delayed degradation of JAK2 protein. Int.J.Cancer.

[10] Guiter C, Dusanter-Fourt I, Copie-Bergman C, Boulland ML, Le GS, Gaulard P, Leroy K, Castellano F (2004). Constitutive STAT6 activation in primary mediastinal large B-cell lymphoma. Blood.

[11] De Leval L, Ferry JA, Falini B, Shipp M, Harris N L (2001). Expression of bcl-6 and CD10 in primary mediastinal large B-cell lymphoma: evidence for derivation from germinal center B cells?. Am.J.Surg.Pathol.

[12] Malpeli G, Barbi S, Moore PS, Scardoni M, Chilosi M, Scarpa A, Menestrina F (2004). Primary mediastinal B-cell lymphoma: hypermutation of the BCL6 gene targets motifs different from those in diffuse large B-cell and follicular lymphomas. Primary mediastinal B-cell lymphoma: hypermutation of the BCL6 gene targets motifs different from those in diffuse large B-cell and follicular lymphomas.

[13] Palanisamy N, Abou-Elella AA, Chaganti S.R, Houldsworth J, Offit K, Louie D.C, Terayu-Feldstein J, Cigudosa J.C, Rao P.H, Sanger W.G, Weisenburger D.D, Chaganti R.S (2002). Similar patterns of genomic alterations characterize primary mediastinal large-B-cell lymphoma and diffuse large-B-cell lymphoma. Genes Chromosomes.Cancer.

[14] Cattoretti G, Pasqualucci L, Ballon G, Tam W, Nandula SV, Shen Q, Mo T, Murty VV, Dalla-Favera R (2005). Deregulated BCL6 expression recapitulates the pathogenesis of human diffuse large B cell lymphomas in mice. Cancer Cell.

[15] Cerchietti LC, Ghetu AF, Zhu X, Da Silva GF, Zhong S, Matthews M, Bunting KL, Polo JM, Fares C, Arrowsmith CH, Yang SN, Garcia M, Coop A, Mackerell AD, Prive GG, Melnick A (2010). A small-molecule inhibitor of BCL6 kills DLBCL cells in vitro and in vivo. Cancer Cell.

[16] Polo JM, Dell'Oso T, Ranuncolo SM, Cerchietti L, Beck D, Da Silva GF, Prive GG, Licht JD, Melnick A (2004). Specific peptide interference reveals BCL6 transcriptional and oncogenic mechanisms in B-cell lymphoma cells. Nat.Med.

[17] Kolonic SO, Dzebro S, Kusec R, Planinc-Peraica A, Dominis M, Jaksic B (2006). Primary mediastinal large B-cell lymphoma: a single-center study of clinicopathologic characteristics. Primary mediastinal large B-cell lymphoma: a single-center study of clinicopathologic characteristics.

[18] Moller P, Bruderlein S, Strater J, Leithauser F, Hasel C, Bataille F, Moldenhauer G, Pawlita M, Barth TF (2001). MedB-1, a human tumor cell line derived from a primary mediastinal large B-cell lymphoma. MedB-1, a human tumor cell line derived from a primary mediastinal large B-cell lymphoma.

[19] Nacheva E, Dyer MJ, Metivier C, Jadayel D, Stranks G, Morilla R, Heward JM, Holloway T, O'Connor S, Bevan PC (1994). B-cell non-Hodgkin's lymphoma cell line (Karpas 1106) with complex translocation involving 18q21.3 but lacking BCL2 rearrangement and expression. Blood.

[20] Sambade C, Berglund M, Lagercrantz S, Sallstrom J, Reis RM, Enblad G, Glimelius B, Sundstrom C (2004). U-2940, a human B-cell line derived from a diffuse large cell lymphoma sequential to Hodgkin lymphoma. U-2940, a human B-cell line derived from a diffuse large cell lymphoma sequential to Hodgkin lymphoma.

[21] Goenka S, Kaplan MH (2011). Transcriptional regulation by STAT6. Transcriptional regulation by STAT6.

[22] Dent AL, Shaffer AL, Yu X, Allman D, Staudt LM (1997). Control of inflammation, cytokine expression, and germinal center formation by BCL-6. Science.

[23] Walker SR, Nelson EA, Frank DA (2007). STAT5 represses BCL6 expression by binding to a regulatory region frequently mutated in lymphomas. STAT5 represses BCL6 expression by binding to a regulatory region frequently mutated in lymphomas.

[24] Pasqualucci L, Migliazza A, Basso K, Houldsworth J, Chaganti RS, Dalla-Favera R (2003). Mutations of the BCL6 proto-oncogene disrupt its negative autoregulation in diffuse large B-cell lymphoma. Blood.

[25] Migliazza A, Martinotti S, Chen W, Fusco C, Ye BH, Knowles DM, Offit K, Chaganti RS, Dalla-Favera R (1995). Frequent somatic hypermutation of the 5' noncoding region of the BCL6 gene in B-cell lymphoma. Proc.Natl.Acad.Sci.U.S.A.

[26] Papadopoulou V, Postigo A, Sanchez-Tillo E, Porter AC, Wagner SD (2010). ZEB1 and CtBP form a repressive complex at a distal promoter element of the BCL6 locus. ZEB1 and CtBP form a repressive complex at a distal promoter element of the BCL6 locus.

[27] Batlle A, Papadopoulou V, Gomes AR, Willimott S, Melo JV, Naresh K, Lam EW, Wagner S.D (2009). CD40 and B-cell receptor signalling induce MAPK family members that can either induce or repress Bcl-6 expression. Mol.Immunol.

[28] Ritz O, Guiter C, Dorsch K, Dusanter-Fourt I, Wegener S, Jouault H, Gaulard P, Castellano F, Moller P, Leroy K (2008). STAT6 activity is regulated by SOCS-1 and modulates BCL-XL expression in primary mediastinal B-cell lymphoma. Leukemia.

[29] Daniel C, Salvekar A, Schindler U (2000). A gain-of-function mutation in STAT6. A gain-of-function mutation in STAT6.

[30] Basso K, Dalla-Favera R (2010). BCL6: master regulator of the germinal center reaction and key oncogene in B cell lymphomagenesis. Adv.Immunol.

[31] Wagner SD, Ahearne M, Ferrigno P.K (2011). The role of BCL6 in lymphomas and routes to therapy. Br.J.Haematol.

[32] Weinstein IB (2002). Cancer. Addiction to oncogenes--the Achilles heal of cancer. Science.

[33] Bentz M, Barth TF, Bruderlein S, Bock D, Schwerer MJ, Baudis M, Joos S, Viardot A, Feller AC, Muller-Hermelink HK, Lichter P, Dohner H, Moller P (2001). Gain of chromosome arm 9p is characteristic of primary mediastinal B-cell lymphoma (MBL): comprehensive molecular cytogenetic analysis and presentation of a novel MBL cell line. Gain of chromosome arm 9p is characteristic of primary mediastinal B-cell lymphoma (MBL): comprehensive molecular cytogenetic analysis and presentation of a novel MBL cell line.

[34] Schroder AJ, Pavlidis P, Arimura A, Capece D, Rothman PB (2002). Cutting edge: STAT6 serves as a positive and negative regulator of gene expression in IL-4-stimulated B lymphocytes. Cutting edge: STAT6 serves as a positive and negative regulator of gene expression in IL-4-stimulated B lymphocytes.

[35] Scheeren FA, Naspetti M, Diehl S, Schotte R, Nagasawa M, Wijnands E, Gimeno R, Vyth-Dreese FA, Blom B, Spits H (2005). STAT5 regulates the self-renewal capacity and differentiation of human memory B cells and controls Bcl-6 expression. STAT5 regulates the self-renewal capacity and differentiation of human memory B cells and controls Bcl-6 expression.

[36] Constantinescu SN, Girardot M, Pecquet C (2008). Mining for JAK-STAT mutations in cancer. Mining for JAK-STAT mutations in cancer.

[37] Baus D, Nonnenmacher F, Jankowski S, Doring C, Brautigam C, Frank M, Hansmann ML, Pfitzner E (2009). STAT6 and STAT1 are essential antagonistic regulators of cell survival in classical Hodgkin lymphoma cell line. STAT6 and STAT1 are essential antagonistic regulators of cell survival in classical Hodgkin lymphoma cell line.

[38] Elo LL, Jarvenpaa H, Tuomela S, Raghav S, Ahlfors H, Laurila K, Gupta B, Lund RJ, Tahvanainen J, Hawkins RD, Oresic M, Lahdesmaki H, Rasool O, Rao KV, Aittokallio T, Lahesmaa R (2010). Genome-wide profiling of interleukin-4 and STAT6 transcription factor regulation of human Th2 cell programming. Immunity.

[39] Wei L, Vahedi G, Sun H.W, Watford WT, Takatori H, Ramos H.L, Takahashi H, Liang J, Gutierrez-Cruz G, Zang C, Peng W, O'Shea JJ, Kanno Y (2010). Discrete roles of STAT4 and STAT6 transcription factors in tuning epigenetic modifications and transcription during T helper cell differentiation. Immunity.

[40] Ritz O, Guiter C, Castellano F, Dorsch K, Melzner J, Jais JP, Dubois G, Gaulard P, Moller P, Leroy K (2009). Recurrent mutations of the STAT6 DNA binding domain in primary mediastinal B-cell lymphoma. Recurrent mutations of the STAT6 DNA binding domain in primary mediastinal B-cell lymphoma.

[41] Hartatik T, Okada S, Okabe S, Arima M, Hatano M, Tokuhisa T (2001). Binding of BAZF and Bc16 to STAT6-binding DNA sequences. Biochem.Biophys.Res.Commun.

[42] Steidl C, Gascoyne RD (2011). The molecular pathogenesis of primary mediastinal large B-cell lymphoma. The molecular pathogenesis of primary mediastinal large B-cell lymphoma.

[43] Weinstein IB (2000). Disorders in cell circuitry during multistage carcinogenesis: the role of homeostasis. Carcinogenesis.

[44] Weinstein IB, Joe AK (2006). Mechanisms of disease: Oncogene addiction--a rationale for molecular targeting in cancer therapy. Mechanisms of disease: Oncogene addiction--a rationale for molecular targeting in cancer therapy.

[45] Michor F, Polyak K (2010). The origins and implications of intratumor heterogeneity. The origins and implications of intratumor heterogeneity.

[46] Ichim CV, Wells RA (2006). First among equals: the cancer cell hierarchy. First among equals: the cancer cell hierarchy.

[47] Reya T, Morrison SJ, Clarke MF, Weissman IL (2001). Stem cells, cancer, and cancer stem cells. Stem cells, cancer, and cancer stem cells.

[48] Almendro V, Marusyk A, Polyak K (2013). Cellular heterogeneity and molecular evolution in cancer. Annu.Rev.Pathol.

[49] Lennerz JK, Kim SH, Oates EL, Huh WJ, Doherty JM, Tian X, Bredemeyer AJ, Goldenring JR, Lauwers GY, Shin YK, Mills JC (2010). The transcription factor MIST1 is a novel human gastric chief cell marker whose expression is lost in metaplasia, dysplasia, and carcinoma. The transcription factor MIST1 is a novel human gastric chief cell marker whose expression is lost in metaplasia, dysplasia, and carcinoma.

[50] Ushmorov A, Ritz O, Hummel M, Leithauser F, Moller P, Stein H, Wirth T (2004). Epigenetic silencing of the immunoglobulin heavy-chain gene in classical Hodgkin lymphoma-derived cell lines contributes to the loss of immunoglobulin expression. Epigenetic silencing of the immunoglobulin heavy-chain gene in classical Hodgkin lymphoma-derived cell lines contributes to the loss of immunoglobulin expression.

[51] Livak KJ, Schmittgen TD (2001). Analysis of relative gene expression data using real-time quantitative PCR and the 2(-Delta Delta C(T)) Method. Methods.

[52] Pfaffl MW (2001). A new mathematical model for relative quantification in real-time RT-PCR. Nucleic Acids Res.

