# Supplementary material for: STAT6-mediated BCL6 repression in primary mediastinal B-cell lymphoma (PMBL)
Source: Oncotarget. 2013 Jul 13;4(7):1093–102. doi: 10.18632/oncotarget.1149 (PMC3759668; doi:10.18632/oncotarget.1149)
Supplement: Supplementary file 1 [file oncotarget-04-1093-s001.pdf]

STAT6-mediated BCL6 repression in primary mediastinal B-cell lymphoma (PMBL) – Ritz et al

Material and Methods

PMBL cases and ethical approval. The PMBL cases were identified retrospectively as part of the biobank Ulm (Institute of Pathology) and the comprehensive cancer center Ulm (CCCU). These cases were anonymized and approval from the local ethics committee was obtained

Immunolabeling (IHC).

BCL6 and/or pSTAT6 were stained on formalin-fixed paraffin- embedded (FFPE) sections using previously established protocols. Briefly, antigen retrieval consisted of boiling in Tris-EDTA buffer at pH9.0 (BCL6) or pH6.0 (pSTAT6) for 20min using a steamer prior to incubation with either anti-BCL6 antibody (#M7211; 1:20; Dako, Aachen, Germany) or anti-pSTAT6 antibody (Tyr641; #cs-9361 at 1:30; New England Biolabs, Frankfurt am Main, Germany) for 1h at room temperature. Dako real detection system alkaline phosphatase/RED (K5005, Dako) and hematoxylin counterstaining were applied for visualization.

Double immunofluorestence (IF).

FFPE sections of cell pellets or snap frozen patient tumor samples (fixed for 10 min in 4% formalin) were stained using established protocols. Briefly, after antigen retrieval (see above), anti-rabbit-Cy3 (#111-165-144, 1:800, Dianova, Hamburg, Germany) and anti-mouse-biotin/streptavidin-AlexaFlour488 (#115-066-062/#S11223, 1:400/1:800, Dianova) were combined with nuclear DAPI counterstain for visualization. In patient samples, B-cell origin of neoplastic cells was demonstrated by labeling cells with CD19 antibody (#M7286, 1:100, Dako).

Image processing and quantification.

Images were taken at x400 using an ISIS Imaging System (v5.3, MetaSystems, Altlußheim, Germany) connected to an Axioskop microscope (Zeiss, Oberkochen, Germany). BCL6 and pSTAT6 markers were evaluated in conjunction with DNA labeling using a quantitative image analysis. We performed quantification by using digital image processing via ImageJ and Photoshop according to previously established semiautomatic protocols using AutoIT (version 3.2.12.0 by Jonathan Bennett). We captured a total of 60 images (3 fields per each of the 10 primary cases and 10 fields per each of the 3 PMBL cell lines) and quantified each marker within the nuclear compartment. Extracted nuclei were assigned to one of four categories based on their nuclear labeling pattern: pSTAT6+/BCL6-, pSTAT6-/BCL6+, pSTAT6+/BCL6+, or pSTAT6-/BCL6-. For comparisons, we expressed the raw nuclear counts as a fraction of total stained nuclei per category to the total number of stained nuclei in each image (Figure 1).

Cell culture, transfections and treatment conditions.

PMBL cell lines MedB-1 and Karpas1106 (K1106) were cultured as described previously [28]. PMBL cell line U-2940 (Leibniz Institute DSMZ, Braunschweig, Germany) was cultured according to established protocols [20]. Cells were transfected with either control siRNA (siCo; #VC300A2; Sigma- Aldrich, Taufkirchen, Germany) or siRNA targeting

STAT6 (#STAT6VHS41762, Life Technologies, Darmstadt, Germany). Nucleofection (0.2nmol per sample) was performed with Amaxa nucleofector using buffer “T” (#VCA1002; Lonza, Cologne, Germany) program N-20 for K1106, buffer “T” (#VCA1002; Lonza) program X-05 for U- 2940, and buffer “V” (#VCA1003; Lonza) program R-01 for MedB-1. Cells were harvested 72h post transfection for all further experiments. Ectopic expression of STAT6VT [29] was performed using 1µg of pcDNA3.1-control plasmid (Life Technologies) or pcDNA3.1 plasmid containing STAT6 sequence with activated mutations (pcDNA3.1 ST6VT) using Amaxa buffer “V”, program U-01 for MedB-1, buffer “T”, program O-20 for K1106; buffer “T”, program X-05 for U-2940. For all subsequent experiments, cells were harvested 48h after transfection. Luciferase reporter assays were described previously [28;40]. Briefly, 4x106 K1106 cells were transfected with siCo or siSTAT6 and after 48h incubation cells were additionally transfected with luciferase reporter constructs. pGL3- basic vector was obtained from Promega (Mannheim, Germany). BCL6 short (BS) construct (-165bp to +436bp), containing BCL6 promoter region and first untranslated exon in front of luciferase reporter gene, was prepared by cloning of PCR product (primers for amplification (5’-3’) (F)gcactccccctcttatgtca (R)gaaaacttggagccaaagca) into pGL3 basic vector. BCL6 long (BL) construct was previously described [26].

Western blot, band shift-, and super shift assays.

Western blot, band shift-, and super shift assays were performed as previously described [28;40]. Briefly, for western blot we used 10µg total extracts per lane and antibodies against BCL6 (N3 #sc-858 at 1:1000; Santa Cruz, Heidelberg, Germany); STAT6 (M-200 #sc-1698 at 1:5000, Santa Cruz); pSTAT6 (Tyr641; #cs-9361 at 1:1000; New England Biolabs); and b-actin as loading control (clone AC-74 at 1:5000; Sigma-Aldrich). For band shift- and super shift assays we used 1µg nuclear extract and 1µg anti-STAT6 (#sc-621, S-20X; Santa Cruz) antibody. DNA probes, representing GAS sites within the BCL6 promoter were prepared by P32-labeling of annealed single oligonucleotides (5’-3’):

alpha (F)aggctcaaattccgagaattgagc, (R)agggctcaattctcggaatttgag;

beta/gamma (F)aggtgttgattcttagaactgggg, (R)aggccccagttctaagaatcaaca;

delta (F)aggaagaagtttctaggaaaggc, (R)agggcctttcctagaaacttctt;

epsilon (F)aggctggaattctcagaactaatt, (R)aggaattagttctgagaattccag

Chromatin immunoprecipitation (ChIP).

The ChIP Assay kit (#17-295, Millipore, Schwalbach, Germany) was used according to the manufacturer's dedicated protocol. For IP pre-cleared chromatin samples were incubated with 1µg antibodies against STAT6 (sc#1698, 1µg, M-200, Santa Cruz). For controls, the antibody was either replaced by phosphate buffered saline or 1µg PTP1B antibody (clone AE4-2J, #PH02; Merck, Darmstadt, Germany). The precipitated gDNA was amplified using primers located within the BCL6 regulatory region (5’-3’): (F)aacctctcgctccctttgt and (R)ggcagcaacagcaataatca. Chromatin was used at 1:10 dilution as a positive PCR-control.

Quantitative PCR.

Total RNA (2µg) was reverse transcribed into cDNA using Superscript II kit (Life Technologies) and amplified using the SYBR Green master mix (BioRad Laboratories,

Munich, Germany) on an iCycler (BioRad). Each PCR reaction was performed in triplicate and average Ct values were calculated using the 2(-DDC(t)) method [51]. Gene expression levels are provided relative to the reference genes (ACTB, HPRT1, GAPDH, RPL13A) analyzed by geNorm software (http://medgen.ugent.be/~jvdesomp/genorm/ ;(last accessioned Nov 2011) 25. Primer sequences (5'-3'; annealing temperature 60°C): BCL6 (F)agagcccataaaacggtcct, (R)agtgtccacaacatgctcca; TP53(F)ctttgaggtgcgtgtttgtg, (R)tcttgcggagattctcttcc; PRDM- 1(F)acatgaccggctacaagacc (R)ggcattcatgtggcttttct, CDKN1A(F) gcagaccagcatgacagattt, (R) ggattagggcttcctcttgga FCER2(F) ttcgagctgaacagcagaga, (R)gacacctgcaactccatcct, RPL13A(F)cggaccgtgcgaggtat, (R)caccatccgctttttcttgtc; HPRT1(F)tgctcgagattgaagg, (R)tcccctgttggtcatt; GAPDH(F)gacacctgcaactccatcct, (R)gccaaaagggtcatcatctc; ACTB(F)tgtggcatccacgaaactac, (R)ggagcaatgatcttgatcttca.

Small molecule inhibition and cell viability assays.

Specific inhibitors targeting BCL6 (79-6) [15] and JAK2 (TG101348, TG) [7] were used at final concentration 250µM and 1µM, respectively. Briefly, 10^6 cells per sample were plated in 24 well plate and inhibitor(s)/DMSO were added once. The cell viability was calculated using trypan blue exclusion method in 24h increments. After 72h the medium (containing inhibitors or DMSO only) was replaced by medium without inhibitors or DMSO only and samples were incubated for an additional week (timepoint 10d). Data are displayed as viable cells relative to DMSO.

Statistical analysis. Student's t-test was used to assess sample differences, and $p<0.05$ was defined as statistically significant. For statistical comparisons we used t- tests, ANOVA and Fisher's exact test. Statistical significance was defined as $p <0.05$.
